# Photobiomodulation and Inorganic Bovine Bone in Guided Bone Regeneration: Histomorphometric Analysis in Rats

**DOI:** 10.3390/jfb14050281

**Published:** 2023-05-18

**Authors:** Nicole Rosa de Freitas, Luísa Belluco Guerrini, Luis Augusto Esper, Michyele Cristhiane Sbrana, Caroline Chepernate Vieira dos Santos, Ana Lúcia Pompéia Fraga de Almeida

**Affiliations:** 1 Postgraduate Program, Bauru School of Dentistry, University of São Paulo, Bauru 17012-901, Brazil; nicolefreitas@usp.br (N.R.d.F.);; 2 Periodontics Sector, Hospital for Rehabilitation of Craniofacial Anomalies, University of São Paulo, Bauru 17012-900, Brazil; 3 Postgraduate Program, Hospital for Rehabilitation of Craniofacial Anomalies, University of São Paulo, Bauru 17012-901, Brazil; 4 Department of Prosthodontics and Periodontics, Bauru School of Dentistry, University of São Paulo, Bauru 17012-901, Brazil

**Keywords:** bone transplantation, lasers, regeneration

## Abstract

The objective of this study was to evaluate the efficacy of photobiomodulation in the bone regeneration of critical-sized defects (CSD) filled with inorganic bovine bone associated or not with collagen membranes. The study has been conducted on 40 critical defects in the calvaria of male rats, divided into four experimental groups (*n* = 10): (1) DBBM (deproteinized bovine bone mineral); (2) GBR (DBBM+collagen membrane); (3) DBBM+P (DBBM+photobiomodulation); and (4) GBR+P (GBR+photobiomodulation). At 30 days postoperative, the animals were euthanized, and after the tissue had been processed, histological, histometric, and statistical analyses were performed. The analyses have taken into account newly formed bone area (NBA), linear bone extension (LBE), and residual particle area (RPA) as variables. The Kruskal-Wallis test has been performed, followed by the Dwass-Steel-Critchlow-Fligner test for comparison between groups (*p* < 0.05). When the DBBM+P group was compared to the DBBM group, it was possible to observe significant statistical differences in all the variables analyzed (*p* < 0.05). The application of photobiomodulation in guided bone regeneration (GBR+P) has shown a decrease in the median value for the RPA variable (26.8) when compared to the GBR group (32.4), with a significant statistical difference; however, for NBA and LBE, the therapy has not provided significant results.

## 1. Introduction

Although autogenous bone is considered the gold standard in reconstructive surgeries due to its osteogenic, osteoinductive, and osteoconductive properties [1], its lack of antigenicity, natural structure, and the presence of type I collagen [2] present some disadvantages, such as high morbidity and uncontrolled resorption rates, which can negatively impact postoperative results [3,4].

Thus, biomaterials are widely used as an alternative to autogenous bone in post-extraction dental alveoli, angular bone defects in teeth, maxillary sinus lift, alveolar ridge augmentation, and other procedures. Ideally, they should be easily handled during the surgical procedure, and they should also have antigenic characteristics, biocompatibility, sterilizability, and space maintainability [4].

Biomaterials may come from various origins. Among them, xenogen, especially Bio-Oss^®^, is the material with the most scientific evidence [3,5]. These materials are derived from bovine bone, whose organic portion is eliminated during laboratory processing in order to avoid rejection after implantation in the receptor bed. After this procedure, the material loses its osteoinductive capacity and only retains its osteoconductive features [3]. However, the original structureof the bone is maintained [4], with similar macro and micropores to those of human spongy bone, to provide an excellent bone structure for the formation of new bone [3]. There are disagreements in the literature regarding the potential for resorption of these substitutes. Thus, given the absence of osteogenic properties and the potential for uncontrolled resorption of the biomaterials currently used, in addition to the delay in repair, photobiomodulation has been widely researched [6,7,8,9,10,11,12,13,14,15].

The use of biological membranes in guided bone regeneration (GBR) provides stabilization of the grafting material in the dependencies of the bone defect as well as excluding epithelial migration towards its interior, maintaining space for osteogenic cells of the guide bone to repopulate the area of the host defect and accelerate bone repair [16,17,18].

By associating photobiomodulating therapy with inorganic bovine bone grafts, the effects related to collagen synthesis and hydroxyapatite result in an improvement in bone repair [9,13,19]. Photobiomodulation (PBM) may increase the expression of bone matrix proteins, accelerating all phases of bone formation, including the inflammatory phase, with a reduction in its infiltrate, improvement in periosteum development, and a significant increase in the formation of the trabecular matrix. In addition to it, precocious deposition of osteocalcin and osteopontin on the edges of the defect in rats irradiated by laser provides a faster and more organized bone repair process [20].

Although many studies have used photobiomodulation, there is no consensus as to the protocol to be used. The aim of this study was to evaluate the effect of this therapy, using a dose of 6 J in an intraoperative application, on the bone regeneration of critical sized defects (CSD) filled with inorganic bovine bone associated or not with collagen membranes.

## 2. Materials and Methods

### 2.1. Experimental Model

**Figure 1:**
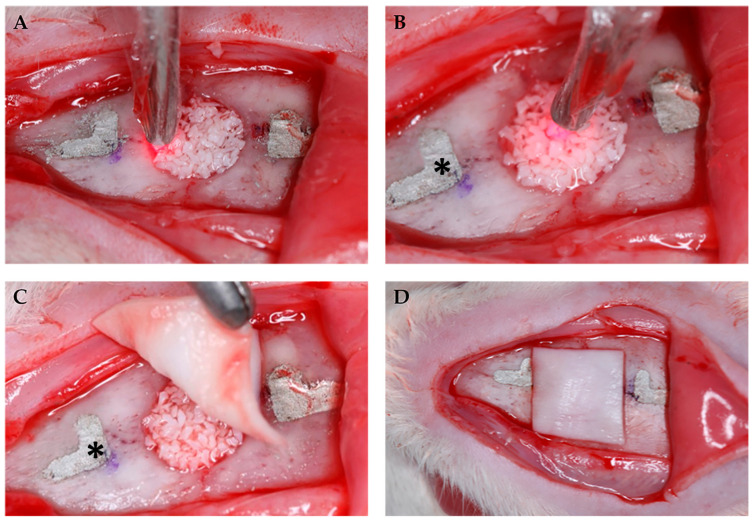
(**A**) Application of photobiomodulation therapy at the edges of the surgical defect and (**B**) in the central point on the bone graft; (**C**) placement of the collagen membrane after photobiomodulation; (**D**) positioned collagen membrane. * L-shaped marks filled with amalgam.

The sample consisted of 40 male rats (*Rattus norvegicusalbinus*, Wistar) from the Central Animal Laboratory of the Bauru School of Dentistry—Bauru, São Paulo, Brazil, weighing between 250 and 300 g and randomly divided into four experimental groups (*n* = 10). Critical size defects (CSD) of 5 mm in diameter have been performed surgically in the calvaria using a trephine bur (JJGC Indústria e Comércio de Materiais Dentários S.A., Curitiba, PR, Brazil), at low speed, under thorough cooling with sterile saline. Extreme care has been taken to protect the dura mater and brain during the surgical procedure [7]. After that, the defects were filled in according to treatment groups. For the experimental procedures, the rats have been anesthetized by an intramuscular injection of ketamine hydrochloride (Vetbrands, Paulínia, Brazil) (0.4 mL/kg) and xylazine (Vetbrands, Paulínia, Brazil) (0.02 mL/kg). The surgical procedure has been performed by only one operator, as follows: (1) antisepsis and trichotomy of the calvaria of each animal; (2) semilunar incision on the calvaria with a full-thickness flap raised; (3) confection of L-shaped marks 2 mm anteriorly and 2 mm posteriorly to the surgical defect margins with conical carbide bur FG-700 (Microdont Micro Usinagem de Precisão Ltda., São Paulo, Brazil); and (4) filling the L-shaped marks with amalgam (the marks are possible to be observed in Figure 1). These marks have been used to identify the center of the original surgical defect during laboratory processing as well as to locate the original bone margins during histometric analysis [7,8,14,15].

After creating the defects, each animal received its respective treatment according to the randomization performed prior to the surgical procedure: (1) DBBM Group (deproteinized bovine bone mineral, Bio-Oss^®^, 0.25–1 mm; Geistlich Pharma AG, Wolhusen, Switzerland); (2) GBR (deproteinized bovine bone mineral + collagen membrane, BioGide^®^, Geistlich Pharma AG, Wolhusen, Switzerland); (3) DBBM+P Group (deproteinized bovine bone mineral + photobiomodulation); (4) GBR+P Group (deproteinized bovine bone mineral + photobiomodulation + collagen membrane).

The flap was repositioned and sutured with silk suture 4-0 (Ethicon, Johnson and Johnson do Brasil Indústria e Comércio de Produtos para Saúde Ltda., São José dos Campos, Brazil), and each animal received a dose of antibiotic (intramuscular injection of 24,000 units of penicillin G-benzathine, Fort Dodge^®^ Saúde Animal Ltda., Campinas, São Paulo, Brazil) [7] and anti-inflammatory (intramuscular dose of 0.3 mg/kg of Ketoprofen, Laboratório Teuto Brasileiro S.A., Anápolis, Goiás, Brazil).

During the period of the study, the rats were kept in an environment with a 12-h light cycle and a temperature between 22 and 24 °C and fed with selected solid food and water ad libitum.

### 2.2. Photobiomodulation

In the DBBM+P group, the defect had been previously filled by the bone graft, and photobiomodulation was performed. In the GBR+P group, after filling the defect and performing photobiomodulation, the collagen membrane was positioned and the flap was sutured. (Figure 1).

**Figure 2:**
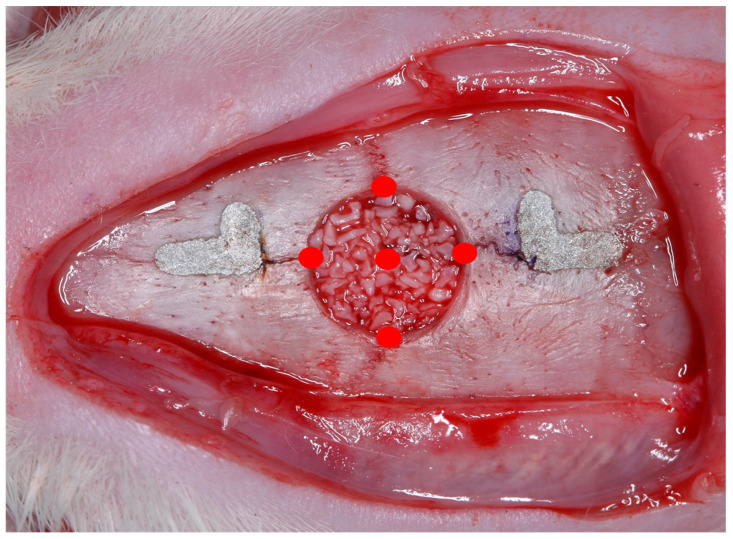
Scheme of application of photobiomodulatory therapy at the edges of the surgical defect in addition to a central point on the bone graft.

Photobiomodulation has been performed in a single application during the transoperative period with a gallium-aluminum-arsenide laser (GaAlAs) (TheraLase DMC^®^, São Carlos, São Paulo, Brazil), in continuous emission mode, with a beam area of 0.028 cm^2^, a 730 nm wave length, 100 mW power, 210 J/cm^2^ energy density, 6 J per point, and an irradiation time of 60 s [15], at four points at the edges of the surgical defect created (12 h, 3 h, 6 h, and 9 h), in addition to a central point on the bone graft [7] (Figure 2).

### 2.3. Tissue Processing

At 30 days postoperative, the animals were euthanized with an excessive dose of anesthetic (5 mg/mL of ketamine hydrochloride and xylazine). The original defect area and the surrounding tissues were removed en bloc, the specimens were processed, and they were longitudinally divided exactly along the center of the CSD, using the L-shaped marks filled with amalgam as a reference. Serial longitudinal cuts were made at 6 µm thick. The specimens were stained using the technique of hematoxylin and eosin (H.E.) for analysis in light microscopy. The tissue processing has been described in detail in previous studies carried out by the group [7,8,14,15].

### 2.4. Histomorphometric Analysis

Images from histological sections representing the central area of the original surgical defect have been captured by a SPOT RT3-2540 Color Slider 2.0 Mp camera (SPOT ImagingSolutions, Diagnostic Instruments, Inc., Sterling Heights, MI, USA) coupled to an Olympus BX50 microscope (Olympus Corporation, Tokyo, Japan) with a 4× amplification and saved in a computer. With the magnification that has been used, it was not possible to capture the entire calvaria in the same image, so a digital image was created by combining three smaller images based on reference structures (blood vessels, bone trabeculae, and particles of grafted material) in each histological section.

Histometric analysis has been performed using a computer image evaluation system, ImageLab 2000 software (Diracon Bio Informática Ltda., Vargem Grande do Sul, São Paulo, Brazil), following the criteria adopted by de Almeida et al. [7] and Cunha et al. [8].

**Figure 3:**
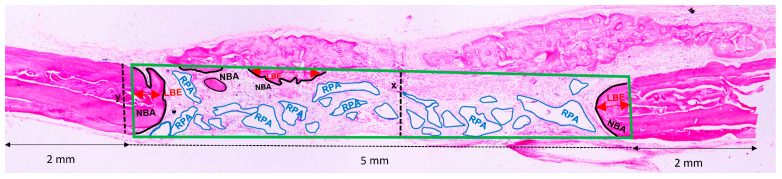
Photomicrograph of a histological section (4x—hematoxylin and eosin) to outline the measurements performed. TA, delimited by the green line, corresponds to the height of the calvaria where the defect has been surgically created. The TA height (x) corresponds to the thickness of the original calvaria bone (y). The NBA was delimited by the black line, the RPA by the blue line, and the LBE by the red arrow.

For each image, the area corresponding to the region of the calvaria bone where the defect had been originally created was delimited and measured in mm^2^, representing 100% of the analyzed area (total area—TA). Taking into account the total length of the specimen of 9 mm and 2 mm as measured from each end to determine the limits of the original surgical defect (5 mm), the areas of newly formed bone area (NBA), residual particle area (RPA) of the implanted materials [7,8,14,15], and linear bone extension (LBE), measured in mm^2^ and calculated as percentages, have been delimited within the TA (Figure 3). 

### 2.5. Statistical Analysis

For each animal, the NBA, LBE, and ARP values have been represented by the arithmetic mean of the four most central sections of the calvaria. The NBA variable has not passed the normality test, so the Kruskal-Wallis test has been performed, followed by the Dwass-Steel-Critchlow-Fligner test for comparison between groups. The results were considered statistically significant when *p* < 0.05. 

## 3. Results

During laboratory processing, one GBR specimen has been lost. 

### 3.1. Qualitative Histological Analysis

**Figure 4:**
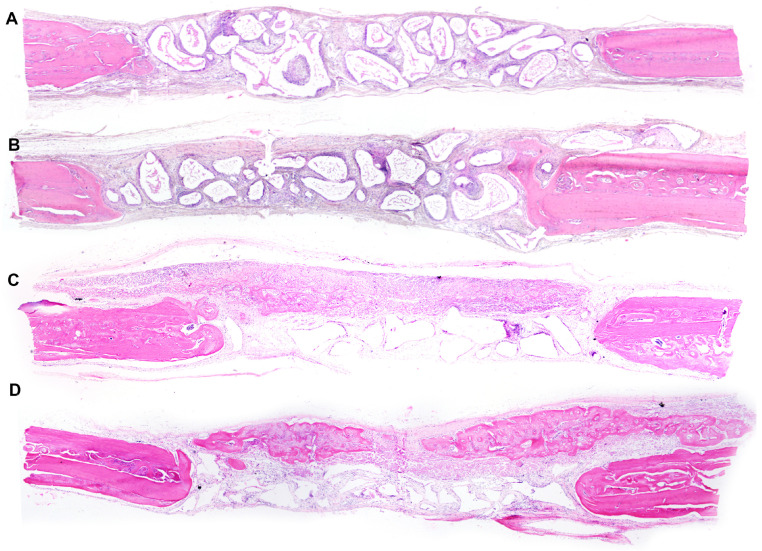
Photomicrographs of panoramic views of surgical defects (4×—hematoxylin and eosin). (**A**)—DBBM Group; (**B**)—DBBM+P Group; (**C**)—GBR Group; (**D**)—GBR+P Group.

The original thickness of the calvaria has been maintained in all groups, but with variable amounts of new bone formation (Figure 4).

**Figure 5:**
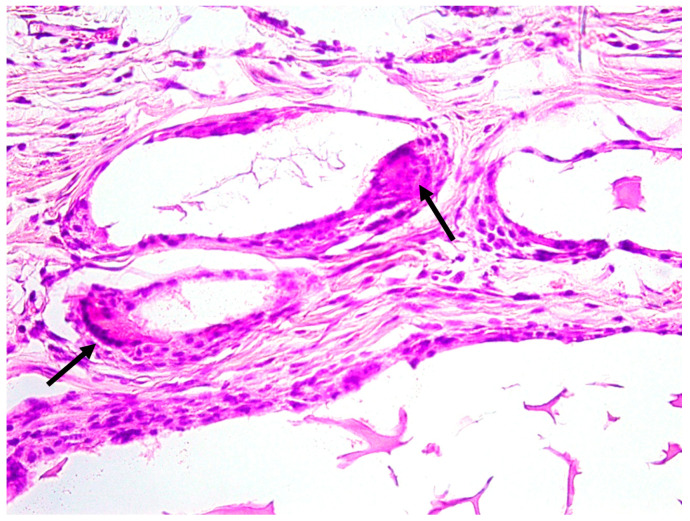
Photomicrograph of the DBBM group (H.E. 40×). Osteoclast on the bovine bone periphery (arrow).

The DBBM group presented minor bone formation at the end of the defect that did not extend to the center in any specimen. In some specimens, the presence of osteoclasts was observed in close contact with residual particles, and the connective tissue was well organized (Figure 5).

**Figure 6:**
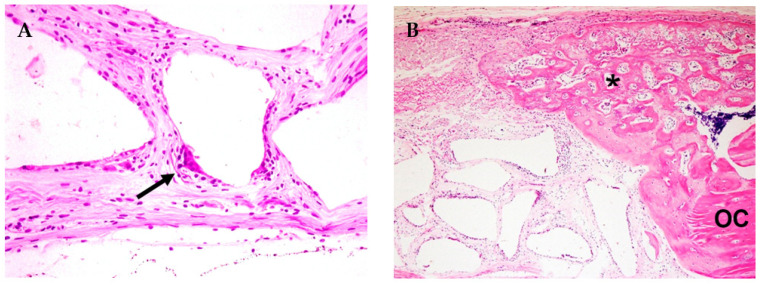
Photomicrograph of the GBR group. (**A**) Osteoclast on the bovine bone particle periphery (arrow) (H.E.—40×). (**B**) Ossification of the collagen membrane towards the center of the defect (asterisk *); OC—original calvaria (H.E.—10×).

In the GBR group, bone formation has been observed with variable extension between specimens and membrane ossification towards the center of the defect. Additionally, the presence of residual particles and well-organized connective tissue has been observed (Figure 6).

**Figure 7:**
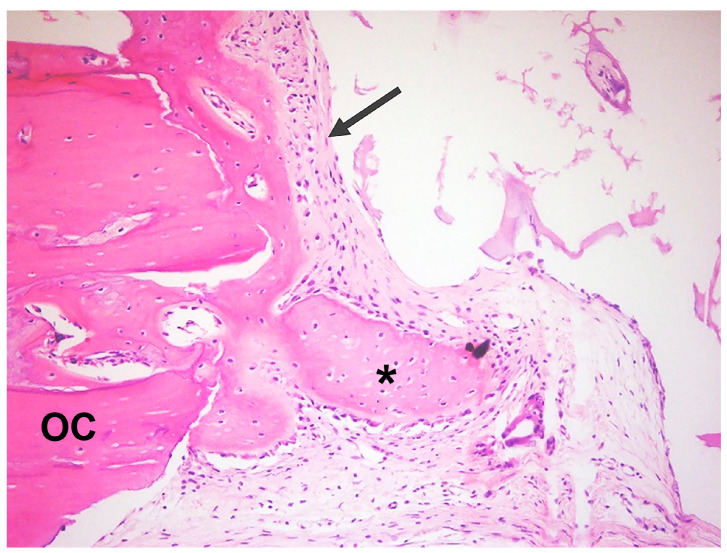
Photomicrographs of the DBBM+P group. Presence of well-organized connective tissue; formation of new bone towards the defect center (asterisk *); and presence of osteoid matrix (arrow); OC—original calvaria (H.E.—10×).

In the DBBM+P group, several specimens have presented new bone formation that extended towards the center of the defect and resulted in in-line closure in three specimens without complete closure in the total area. The presence of residual particles inside the defect allowed maintenance of the thickness of the original calvaria and the beginning of bone formation inside the particles in some specimens, as well as the presence of osteoclasts around them. These remaining particles have been encircled by thick bundles of collagen fibers and the osteoid matrix (Figure 7).

**Figure 8:**
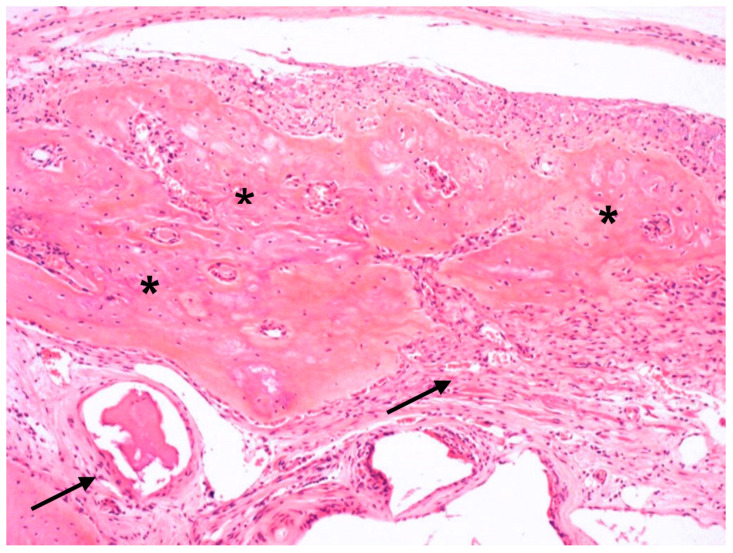
Photomicrograph of the GBR+P group. Ossified collagen membrane (asterisks *); presence of well-organized connective tissue (arrows) (H.E.—10×).

The GBR+P group has displayed membrane ossification above its limits. Furthermore, new bone that extended to the center of the defect has been detected in several specimens without total closure of the defect. The biomaterial particles have been maintained close to the defect, with the presence of surrounding bone formation in some specimens. The connective tissue has been well vascularized, with parallel fibers and several fibroblasts (Figure 8).

### 3.2. Histometric and Statistical Analysis

The analysis using the median, first, and second quartiles is described in Table 1 and in comparison between groups in Table 2.

When the DBBM+P group was compared to the bovine bone used alone (DBBM group), it was possible to observe that the former had higher medians for new bone area (42.2) and linear bone length (79.4). This comparison showed a lower median value for the residual particle area variable (22.4), with a statistically significant difference (*p* < 0.05) in all analyzed variables, while the second had the lowest medians, both for NBA and LBE (9.4 and 23.6, respectively), and the highest median for particle residual area (37.7).

By associating the membrane with the grafting material (GBR), the results show improvement in all variables, with a median of 19.9 for NBA and LBE of 71.9, with a statistically significant difference (*p* = 0.013), and a slight decrease in the median of RPA (32.4), but without a significant difference. The application of photobiomodulatory therapy to these materials (GBR+P group) did not improve the NBA and LBE variables but showed a decrease in the median value of the RPA variable (26.8) when compared to the GBR and the DBBM groups (*p* = 0.017 and *p* = 0.003, respectively) with a significant statistical difference.

## 4. Discussion

The effects of photobiomodulation on critical size defects in rat calvaria treated with bovine bone graft and collagen membrane have been evaluated. Bone formation has been observed in the collagen membrane region that exceeds the limits of the defect originally created. Furthermore, the therapy has shown positive results on demineralized bovine bone and provided greater bone formation in new bone areas while accelerating the resorption of residual particles. 

The 30-day analysis has been carried out due to the fact that photobiomodulation acts in the initial phases of regeneration. At this period, there are a large number of differentiating cells, while in late stages, these cells decrease in number and therefore may reduce the effectiveness of the PBM in stimulating bone formation [21].

There are discrepancies about the ideal photobiomodulation protocol. Several investigators have evaluated the effectiveness of various low-intensity lasers, including GaAlAs, at different wavelengths, doses, output power, and treatment protocols [13,19,21,22,23,24]. The protocol used in the present study was based on previous publications, which used a dose of 6 J in a single application and achieved positive results in CSD regeneration [7,8,14,15]. 

We suggest that an application in the transoperative period, with an ideal amount of energy applied to the edges of the surgical defect and on the bone graft, is sufficient to observe the effects of the photobiomodulatory therapy. The stage at which PBM is used may influence the amount of newly formed bone, suggesting that the mechanism accelerates the process of bone formation in the first stages of healing in which there is greater cellular experience [23], mesenchymal cell differentiation [25], and higher alkaline phosphatase activity (ALP), providing an increase in the number of differentiated cells that express markers of osteoblastic differentiation generated in bone formation [6].

The biostimulatory effect of the laser occurs when, upon reaching the cell, the light is absorbed by specific chromophores in the mitochondria. This provides electron excitation, increases adenosine triphosphate (ATP) production, controls the release of reactive oxygen species, and regulates the production of transcription factors, which are molecules that regulate the activity of other cells, including proliferation, differentiation, and the secretion of growth factors [26]. Although several prior studies have used repeated applications [9,10,12,21,27,28], there are already reports of positive results with a single application [7,8,13,14,15,29].

In order to translate our experiments into clinical practice, a single application during the surgical procedure is believed to facilitate the use of the therapy. Repeated sessions may be possible in research settings and/or universities; however, in daily clinical practice, it may not be that easy [30], as they might increase the time and cost of dental treatment.

The application of therapy to the edges of the defect has been performed in order to stimulate the bone cells at the margins of the defect surgically created. The application of therapy to the grafting material is not clearly shown in the literature [19]. Thus, in an attempt to optimize the results and considering the possibility of therapy improvement of the osteoconductive power of the material, photobiomodulation has been applied to the central point of the defect, over the graft, before positioning the membrane in order to avoid blocking the passage of the light.

In the present study, a positive effect of photobiomodulation on DBBM has been observed. The combination of therapy with bovine bone graft (DBBM+P group) provided better repair, with significant statistical differences among all variables observed (NBA, LBE, and RPA). When the xenogen material is used alone, effects related to collagen and hydroxyapatite synthesis occur more slowly [8,13,22], but when the photobiomodulatory therapy is used in conjunction with the biomaterial, excellent results can be obtained, allowing for greater production of collagen from fibroblasts and osteoid matrix [21]. In addition, PBM can provide organization of collagen fibers, corroborating our histological findings in the DBBM+P and GBR+P groups, which showed better fiber organization when compared to the other groups.

The PBM can increase the osteoconductive potential of the biomaterial and maintain bone volume until the end of the analysis [9]. In addition, it may provide greater expression of the OPG and RANKL biomarkers, indicating that the presence of the biomaterial could induce controlled ossification in surgical defects [13].

Acceleration of the resorption of residual particles enabling the formation of new bone (DBBM+P group) has been observed as a positive effect of PBM, which corroborates the findings of Cunha et al. [8] and Sbrana et al. [15]. This can be explained by photobiomodulation’s actions in stimulating osteoblastic and osteoclastic activity without changing the bone structure [31]. However, our results differ from those of Bosco et al. [22], who did not identify a relationship between the use of low-intensity lasers and residual particle resorption. These may occur due to differences in materials and photobiomodulation protocols between the studies. However, the role of photobiomodulatory therapy on residual particles is still unclear [21].

In all specimens of the study, it was possible to observe the maintenance of calvaria thickness and the presence of biomaterial particles, which were sometimes surrounded by osteoid matrix and other times by osteoclasts. These findings corroborate those of other studies that reported that bovine bone graft granules undergo slow resorption. Notably, instead of being reabsorbed and entering the physiological process of bone resorption, they remained surrounded by newly formed bone [32,33]. This provided a structure for bone formation that kept tissues in position and prevented the resorption of the defect. There are authors who consider Bio-Oss^®^ (BO) non-resorbable and harmful to the repair; once non-resorbable granules are present, they may negatively interfere with the repair of defects and compromise the osseointegration of implants [34,35]. Nonetheless, when extraction sockets were clinically treated with BO, there was bone formation in sufficient quality and quantity for implant installation in the correct position with maintenance of socket thickness [36]. Oliveira et al. [9] found approximately 60% more bone in the control group, but the microtomography of the groups that used bovine bone showed mineralized bone. The presence of osteoconductive material in the defect may even delay the repair and the formation of new bone; however, it may improve bone quality, and it is very important to maintain the bone morphology.

Bosco et al. [22] have stated that regardless of the presence of the biomaterial, photobiomodulation is capable of improving bone formation. In this study, a photobiomodulation group has not been included because the intention was to observe the effect of therapy on the biomaterial. Previous studies conducted by our group have also shown reliable results from the therapy used alone, although there has been no maintenance of the original thickness of the calvaria, which directly affects the results when they are transposed into clinical practice because maintaining the thickness is extremely important for rehabilitation and therefore the success of the implant.

The collagen membrane is used in GBR in order to provide space for the osteogenic cells of the host bone to repopulate the region. It prevents connective tissue cells from migrating to regions near the defect and compromising bone regeneration [16,17]. Its use also provides greater stability for the grafting material in the region near the defect [37].

The association of PBM therapy in GBR could further optimize the results by increasing the concentration of calcium hydroxyapatite, the main substrate for bone structure, which provides greater bone maturation [19,23]. However, in the present study, there was no significant difference related to this association. The histological analysis revealed a greater amount of new bone formation below, above, and inside the collagen membrane in the GBR+P group. In the GBR group, membrane ossification was observed but in smaller proportions, which corroborates the findings of Freitas et al. [14]. Membrane ossification can be explained by the findings of Elgali et al. [37], who observed the presence of different cell phenotypes in the membrane region at different periods of the study, with a high prevalence of osteogenic cells and macrophages/monocytes that migrated to the inside of the membrane. This indicates that instead of the membrane acting as a barrier, it has properties that promote cell migration, cell involvement, and differentiation, regardless of the presence or absence of bone substitutes. Bone neoformation beyond the limits of defects has not been assessed in the present study, which may have influenced the results.

The closure of the defect, expressed by the variable LBE, showed greater bone formation in the DBBM+P group (79.4). This result can be explained by the fact that PBM stimulates vascularization and acts early in the inflammatory phase, favoring bone neoformation [22,29]. This is extremely important in situations where the regenerative challenge is more complex, as in the case of smoking patients or even those who require grafting materials that can delay bone formation [29]. Notably, regardless of the application of a low-intensity laser and the use of a collagen membrane, good bone formation results have been observed. Furthermore, when analyzing histological images, the GBR+P group displayed greater membrane mineralization and bone formation beyond the thickness of the calvaria compared to the GBR group. The NBA and LBE observed beyond the delimitation of the TA were not taken into account in this study due to the methodology employed. Thus, significant statistical differences have not been observed when these groups were compared (GBR vs. GBR+P). However, there have been reports showing that photobiomodulating therapy in conjunction with GBR increases the concentration of calcium hydroxyapatite, the main substrate for bone structure, and provides greater bone maturation [18,19].

In the present study, only the histomorphometric analysis has been performed, which is a limitation of the same. Analyses that take into account bone formation beyond the limits of the defect, as well as immunohistochemistry and microtomography techniques, were necessary to identify the bone markers that were present in bone repair as well as bone density. This is especially important for verifying the potential of therapy to improve bone quality in the groups that received photobiomodulation.

## 5. Conclusions

The results of the present study indicate that photobiomodulation in guided bone regeneration of critical-size defects does not provide greater new bone formation for closuring the defect. However, bone formation has been observed in the region of the collagen membrane that exceeds the limits of the originally created defect. In addition, the therapy has shown positive results on demineralized bovine bone and provided greater bone formation in the area, as well as, to a concomitant extent, the resorption of residual particles.

## Figures and Tables

**Table 1:** Means, standard deviations, medians, Q25, and Q75 values of newly formed bone area (NBA), linear bone extension (LBE), and residual particle area (RPA) in %.

	Group	NBA	LBE	RPA
N	DBBM	10	10	10
	DBBM+P	10	10	10
	GBR	9	9	9
	GBR+P	10	10	10
Mean	DBBM	11.4	27.4	38.7
	DBBM+P	48.6	76.2	16.7
	GBR	22.0	62.9	32.3
	GBR+P	19.0	48.9	24.2
Satandard deviation	DBBM	7.8	15.5	6.9
	DBBM+P	28.2	29.0	15.2
	GBR	10.7	21.6	6.2
	GBR+P	13.7	21.7	6.0
Median	DBBM	9.4	23.6	37.7
	DBBM+P	42.2	79.4	22.4
	GBR	19.9	71.9	32.4
	GBR+P	13.6	57.1	26.8
Q 25	DBBM	6.5	18.7	36.2
	DBBM+P	35.9	51.9	0
	GBR	15.8	48.6	29.9
	GBR+P	9.6	34.1	21.2
Q75	DBBM	11.4	31.1	42.2
	DBBM+P	74.6	96.8	27.5
	GBR	24.6	77.3	37.4
	GBR+P	28.2	62.4	28.3

**Table 2:** Comparison between groups (Dwass-Steel-Critchlow-Fligner test, *p* < 0.05).

	NBA	LBE	RPA
DBBM × DBBM+P	0.017 *	0.005 *	0.008 *
DBBM × GBR	0.068	0.013 *	0.238
DBBM × GBR+P	0.525	0.148	0.003 *
DBBM+P × GBR	0.238	0.761	0.054
DBBM+P × GBR+P	0.106	0.232	0.666
GBR × GBR+P	0.713	0.663	0.017 *

The presence of * indicates a statistically significant difference.

## Data Availability

The data presented in this study are openly available in FigShare at doi 10.6084/m9.figshare.22858028.
